# Impact of Ten-Valent Pneumococcal Conjugate Vaccination on Invasive Pneumococcal Disease in Finnish Children – A Population-Based Study

**DOI:** 10.1371/journal.pone.0120290

**Published:** 2015-03-17

**Authors:** Jukka Jokinen, Hanna Rinta-Kokko, Lotta Siira, Arto A. Palmu, Mikko J. Virtanen, Hanna Nohynek, Anni Virolainen-Julkunen, Maija Toropainen, J. Pekka Nuorti

**Affiliations:** 1 Department of Vaccination and Immune Protection, National Institute for Health and Welfare, Helsinki, Finland; 2 Department of Infectious Disease Surveillance and Control, National Institute for Health and Welfare, Helsinki, Finland; 3 Department for Promotion of Welfare and Health, Ministry for Social Affairs and Health, Helsinki, Finland; 4 Department of Epidemiology, School of Health Sciences, University of Tampere, Tampere, Finland; Centers for Disease Control & Prevention, UNITED STATES

## Abstract

**Background:**

The ten-valent pneumococcal conjugate vaccine (PCV10) was introduced into the Finnish National Vaccination Program (NVP) in September 2010 with a 2+1 schedule (3, 5, 12 months) without catch-up vaccinations. We evaluated the direct and indirect effects of PCV10 on invasive pneumococcal disease (IPD) among children ≤5 years of age during the first three years after NVP introduction.

**Methods:**

We conducted a population-based, observational follow-up study. The cohort of vaccine-eligible children (all children born June 1, 2010 or later) was followed from 3 months of age until the end of 2013. For the indirect effect, another cohort of older children ineligible for PCV10 vaccination was followed from 2011 through 2013. Both cohorts were compared with season- and age-matched reference cohorts before NVP introduction. National, population-based laboratory surveillance data were used to compare culture-confirmed serotype-specific IPD rates in the vaccine target and reference cohorts by using Poisson regression models.

**Results:**

The overall IPD rate among vaccine-eligible children was reduced by 80% (95%CI 72 to 85); the reduction in vaccine-type IPD was 92% (95%CI 86 to 95). However, a non-significant increase in non-vaccine type IPD was observed. During 2012–2013, we also observed a 48% (95%CI 18 to 69) reduction in IPD among unvaccinated children 2 to 5 years of age, which was mostly attributable to the ten vaccine serotypes.

**Conclusions:**

This is the first population-based study investigating the impact of PCV10 introduction without prior PCV7 use. A substantial decrease in IPD rates among vaccine-eligible children was observed. A smaller and temporally delayed reduction among older, unvaccinated children suggests that PCV10 also provides indirect protection against vaccine-type IPD. Changes in serotype distribution warrant continuous monitoring of potential increases in non-vaccine serotypes.

## Introduction

*Streptococcus pneumoniae* (pneumococcus) causes a wide variety of clinical infections from mucosal respiratory infections to invasive pneumococcal disease (IPD) such as meningitis, bacteremia and bacteremic pneumonia. The considerable public health burden affects particularly young children and older adults. Randomized controlled trials have demonstrated that pneumococcal conjugate vaccines (PCVs) are highly efficacious in preventing IPD in children [1]. However, long-term population effects of routine PCV use, such as herd effects, vaccine impact on uncommon serotypes, and changes in serotype distribution, can generally only be demonstrated during widescale use [2,3]. Therefore, observational studies comparing IPD rates before and after introduction of PCV into National Vaccination Programs (NVP) are needed to assess the overall public health impact of PCVs.

The bulk of currently available data on the public health impact of PCVs is based on the 7-valent PCV (PCV7) experience [2,3]. The effectiveness of 10-valent PCV (PCV10) against IPD in vaccinated children was recently demonstrated in two randomized trials [4, 5] but limited evidence is available on its effects during routine use [6, 7, 8] and no population-based studies have reported PCV10 effects in populations without previous PCV use.

As a result of a public tender, the Ministry of Social Affairs and Health decided to introduce PCV10 as the first PCV in the Finnish National Vaccination Program in September 2010. All children born June 1^st^, 2010 or thereafter were eligible for vaccination with a 2+1 schedule (vaccinations at 3, 5, and 12 months of age). No catch-up vaccinations were offered. We evaluated the impact of PCV10 on IPD among vaccine-eligible children and the indirect effects among older, unvaccinated children 2 to 5 years of age.

## Methods

We used a nation-wide, population-based follow-up study design. For total (direct and indirect) effects, we compared IPD rates in the PCV10-eligible cohort to season- and age-matched reference cohorts before vaccine introduction. For indirect effects, we compared IPD rates in a cohort of older children not eligible for vaccination during the PCV10-NVP with season- and age-matched reference cohorts before vaccine introduction.

### Study population and vaccination program

The size of annual birth cohort in Finland (pop. 5.5 million) is approximately 60.000. All newborns are assigned a unique personal identity code at birth, enabling linkage of disease and vaccination records from local and national health databases and registers. The population under study was defined by using data from the Finnish Population Information System, which includes an online record of Personal Identity Code, name, gender, date of birth, and place of residence for all permanent residents in Finland.

Finnish municipalities (local administrative areas, N>300) are responsible for providing primary health care services, including NVP vaccinations. Childhood vaccinations are given at public well-baby (child health) clinics during the first year of life. Details of vaccinations are recorded in electronic primary health care databases. Preliminary estimates of PCV10 coverage were obtained by using data from the recently established National Vaccination Register [9] which collects information on vaccinations from local health care centers.

Before introduction of PCV10 into NVP in 2010, use of PCVs in Finland since the licensure of PCV7 in 2001 and PCV10 and PCV13 in 2009 was minimal. Since 2001, PCV7 was recommended to a small group of children with specific high risk conditions [10]. On the basis of national sales figures (doses distributed), the estimated uptake of PCV7 among children <2 years of age was less than 2% until 2009. During 2009–2010, a large cluster-randomized PCV10 effectiveness trial (FinIP) was conducted in Finland [4], during which approximately 30,000 children <1.5 years of age received PCV10 (20% of the corresponding birth cohort in Finland).

### Disease surveillance and laboratory methods

In Finland, blood and CSF cultures from febrile pediatric patients are taken only at hospital emergency clinics and hospital wards according to clinical guidelines. All clinical microbiology laboratories in Finland are required to notify isolation of *Streptococcus pneumoniae* from blood and/or cerebrospinal fluid (CSF) to the National Infectious Disease Register (NIDR), a population-based, electronic laboratory surveillance system maintained by the National Institute for Health and Welfare (THL) since 1995. Possible multiple pneumococcal notifications concerning the same individual and pathogen within three months from the first notification are merged into a single case [11].

The clinical microbiology laboratories also submit pneumococcal isolates from reported cases to THL reference laboratories for further analysis, such as species verification and serotyping. Both reporting and sending of the isolates is mandated by law. From 1995 through 2009, pneumococcal isolates were serotyped by latex agglutination and/or counterimmunoelectrophoresis supplemented with Quellung reaction, when needed. Since 2010, isolates have been serotyped by multiplex PCR supplemented with Quellung reaction, when needed. A bridging study was conducted to ensure that the results based on the two serotyping methods are comparable [12]. In addition, all serotype 6A cases in the cohorts under study were recently serotyped for the second time to distinguish serotype 6C from 6A cases. No 6C cases were identified among children ≤5 years of age in the study cohorts.

Comprehensive linkage of NIDR notifications and serotyping results using Personal Identity Code has been available since 2004. The completeness of reporting of IPD cases to the NIDR was recently validated in a register study [13], which also showed that all reported pediatric IPD cases were hospitalized and treated as inpatients.

Information employed in this study is based on the above-described surveillance data. Permission to use these register data was applied from and granted by THL (THL/1090/6.02.00/2013) which is the regulative body for granting access to these data for research purposes, and all data were anonymised before use.

### Outcome definition and study design

To compare culture-confirmed IPD rates before and after NVP introduction among vaccine-eligible and unvaccinated children, we constructed PCV10 target and reference cohorts by using the data from the Population Information System. A case of IPD was defined as isolation of *S*. *pneumoniae* by culture from blood or CSF and reported to National Infectious Disease Register. IPD cases with date of specimen from 2004 to 2013 were included. During the study period, isolates were available for serotyping from more than 96% of notified cases. IPD cases were categorized into three mutually exclusive groups: PCV10-types (1, 4, 5, 6B, 7F, 9V, 14, 18C, 19F, 23F), PCV10-related types (remaining types of the same serogroup as PCV10-types) and non-PCV10 types. Diagnosis of meningitis was defined as isolation of *S*. *pneumoniae* from CSF.

### Cohorts and follow-up for assessment of total (direct and indirect) effects

**Fig 1:**
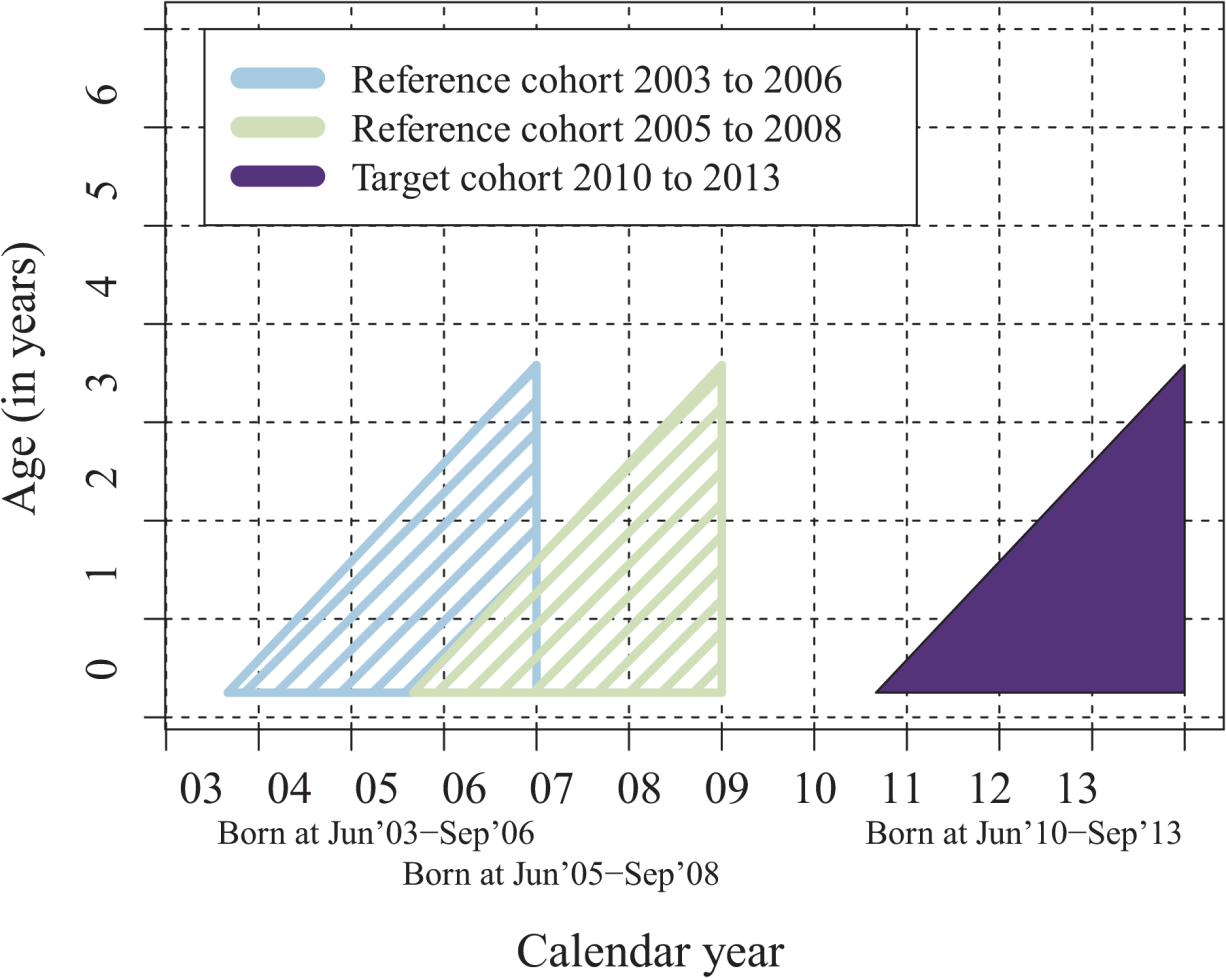
Target and reference cohorts for comparing the total impact of PCV10 on IPD in vaccine-eligible children.

Vaccine-eligible children were defined as those with birthdates from June 2010 to September 2013 (PCV10 target cohort, Fig. 1) regardless whether they had received the vaccine or not. PCV10 uptake among these children was estimated at 95% [9]. For the assessment of total effects of PCV10, we constructed two age- and season-matched reference cohorts which included children born June 2003—September 2006 (Reference cohort 1, Fig. 1), and June 2005—September 2008 (Reference cohort 2, Fig. 1). In each of the cohorts, follow-up started at three months of age (i.e., the expected age at the first dose). To minimize the influence of the FinIP trial vaccinations [4] in the reference cohorts, the cohorts were formed to exclude years 2009–2010 from the follow-up (Fig. 1).

### Cohorts and follow-up for assessment of indirect effects

**Fig 2:**
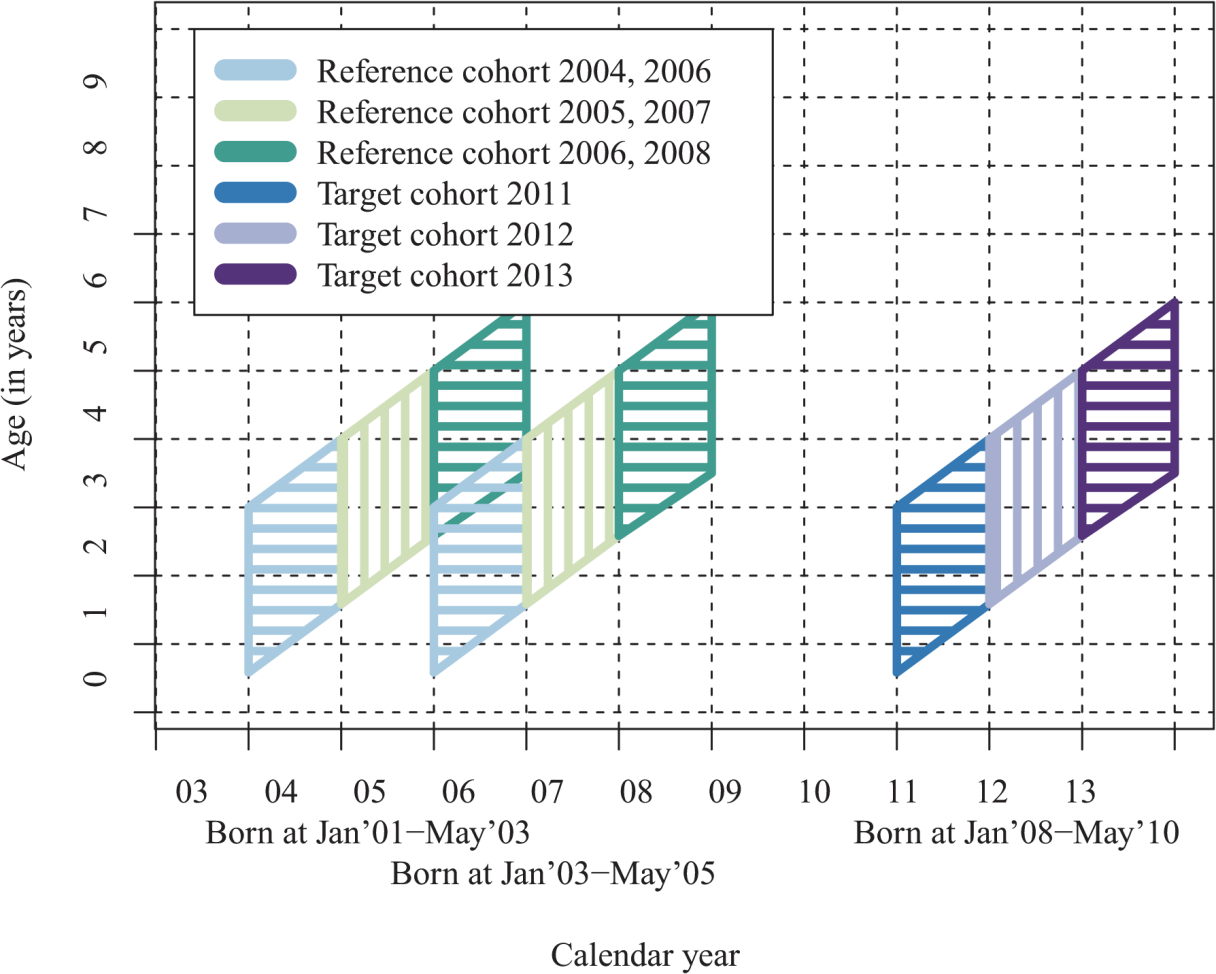
Target and reference cohorts for comparing the indirect impact of PCV10 on IPD in unvaccinated older children.

For the evaluation of indirect PCV10 effects, the unvaccinated cohort of children was defined as those born January 2008—May 2010 (target cohort for indirect effects, Fig. 2). Children vaccinated with PCV10 in the FinIP cluster-randomized PCV10 effectiveness trial [4] were excluded. For the comparison, we constructed two age- and season-matched reference cohorts which included children born January 2001—May 2003, and January 2003—May 2005 (Fig. 2).

In the analysis, the follow-up time was divided into three calendar year periods to assess the timing of the potential indirect effects after PCV10 introduction. In the target cohort, the first period included year 2011, the second period year 2012, and the third period 2013 (Fig. 2). These periods were compared with the age- and season-matched periods in the two reference cohorts (Fig. 2).

### Statistical analysis

IPD rates in the target cohort and combined reference cohorts were compared by using Poisson regression models. Numbers of IPD cases in the cohorts during the observation periods were tabulated and the corresponding person-years of follow-up were used as weights in the analysis. Relative rate reduction (percent) was calculated as (1—relative risk)*100%. In case of zero IPD cases in either cohorts, ratio of Poisson means was derived conditional on total number of cases [14] and Clopper-Pearson confidence interval for the resulting binomial distribution was used [15]. Absolute rate reductions and the corresponding 95% confidence intervals were calculated from the model estimates using the delta method.

In order to obtain a robust baseline and to avoid the impact of short-term secular trends, we used two reference cohorts for calculating baseline rates. Earlier cohorts with follow-up before 2004 were not included (see Figs. 1 and 2) because the Personal Identity Code for linking IPD reports with submitted isolates was not routinely available before 2004.

## Results

**Fig 3:**
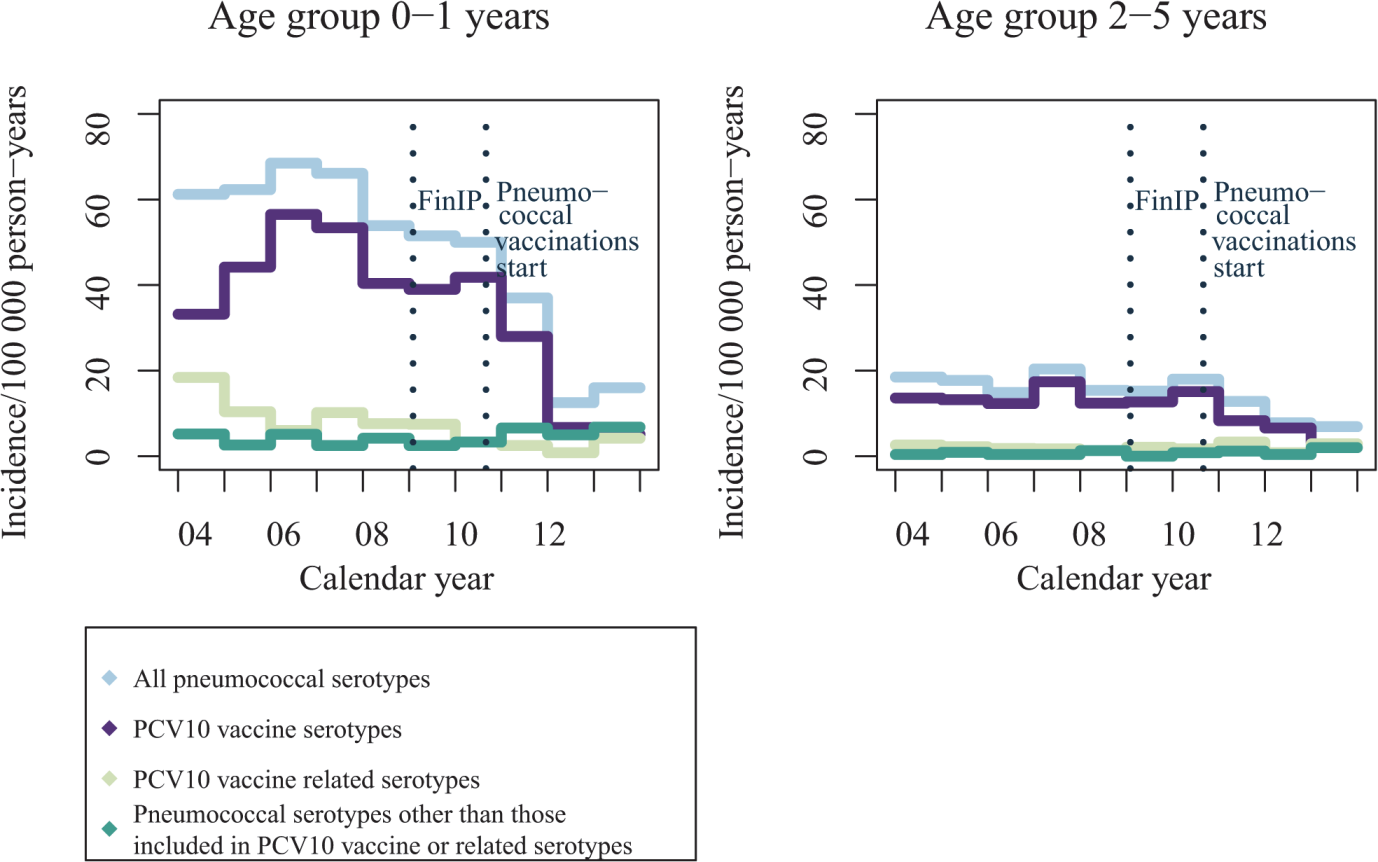
Annual serotype-specific rates of IPD in children <2 years of age and children 2–5 years of age, Finland 2004–2013

During 2004–2008, the annual incidence of IPD in children <2 years of age ranged from 52 to 69 per 100,000 person-years. After introduction of the PCV10-NVP there was a sharp decrease in IPD in this age group. This resulted mainly from a reduction in vaccine serotypes, which had nearly disappeared by 2013 (Fig. 3).

### Total (direct and indirect) effectiveness of PCV10 in children

In the target cohort eligible for vaccination, 334 087 child-years were available for the NVP impact analysis. By the end of follow-up, the children were 3–42 months of age (Fig. 1). Table 1 shows rates of IPD per 100,000 person-years (N cases) in the target cohort eligible for PCV10, and the relative and absolute rate reduction compared with the reference cohorts. The overall decrease in any culture-confirmed IPD following NVP introduction was 80% (95% CI 72 to 85); for PCV10-type IPD it was 92% (95%CI 86 to 95). The reduction in IPD caused by PCV10-related types was 68% (95%CI 38 to 85), whereas non-PCV10 serotypes increased by 2.8 cases per 100,000 person-years (95%CI −0.2 to 5.7). The absolute rate reduction in the overall IPD after vaccination was 50 cases per 100,000 person-years (95%CI 43 to 57). Of the 14 PCV10-type cases in the target cohort, 13 occurred in non-vaccinated or partially-vaccinated children. One case of serotype 19F IPD occurred in a child who had received all three doses of PCV10.

**Table 1:** Rates of IPD and the corresponding rate reductions in the PCV10 eligible target cohort vs. reference cohorts.

Serotype group	Incidence/100 000 person-years (N)		Relative rate reduction (95% CI)	Absolute rate reduction (95% CI)
	Reference cohorts combined^1^ ^)^	Target cohort^2^ ^)^	Target vs. reference cohorts combined	Target vs. reference cohorts combined
PCV10 serotypes^3^ ^)^	49.1 (162+157)	4.2 (14)	92 (86, 95)	44.9 (39, 51)
PCV10-related serotypes^4^ ^)^	8.3 (31+23)	2.7 (9)	68 (38, 85)	5.6 (3, 8)
6A	2.2 (5+9)	0.0 (0)	100 (41, 100)	2.2 (1, 3)
19A	5.5 (23+13)	2.1 (7)	62 (20, 85)	3.4 (1, 6)
Non-PCV10 serotypes^5^ ^)^	3.2 (11+10)	6.0 (20)	-85 (-243, 0)	-2.8 (-6, 0)
3	0.5 (1+2)	2.1 (7)	-354 (-2006, -26)	-1.6 (-3, 0)
22F	0.3 (1+1)	1.2 (4)	-289 (-2707, 24)	-0.9 (-2, 0)
Undefined^6^ ^)^	2.3 (12+3)	0.0 (0)	100 (46, 100)	2.3 (1, 4)
Any culture confirmed IPD	62.9 (216+193)	12.9 (43)	80 (72, 85)	50.1 (43, 57)

1) Follow-up years in the two reference cohorts 649,877, age 3–42 months, born Jun’03-Sep’06 or Jun’05-Sep’08

2) Follow-up years in the target cohort 334,087, age 3–42 months, born Jun’10-Sep’13

3) In these data: 1, 4, 6B, 7F, 9V, 14, 18C, 19F, 23F

4) In these data: 6A, 7C, 9N, 18B, 19A

5) In these data: 3, 10, 10A, 11A, 12F, 15A, 15B, 15C, 22F, 33, 33A/F, 35B, 35F, 38, NC

6) No isolate available

There were 16 and 9 cases of meningitis in the two reference cohorts (6% of all IPD cases). After NVP introduction, a total of 7 meningitis cases were observed in the target cohort eligible for vaccination. Relative rate reduction was 46% (95%CI −19 to 78) and absolute rate reduction 2 cases per 100,000 children per year (95%CI 0 to 4). The number of PCV10-type meningitis cases was 11 and 8 in the reference cohorts, and 3 in the target cohort; relative rate reduction 69% (95%CI 10 to 93).

To investigate temporal effects of NVP-introduction, a sub-analysis was performed comparing IPD-rates in the target cohort during 2013 with the corresponding rates in the reference cohorts. The relative reduction in PCV10-types was 96% (95%CI 89 to 99), but the point estimate for PCV10-related type reduction was smaller (8%, 95%CI −107 to 62) and the increase in non-PCV10 types larger (−135%, 95%CI −457 to −1) compared with the entire post-vaccination follow-up.

### Indirect PCV10 effects in unvaccinated children

The IPD rates per 100,000 person-years during the follow-up years 2011, 2012 and 2013 separately are shown in S1, S2 and S3 Tables, respectively.

Table 2 shows the relative rate reduction in the target cohort (unvaccinated children 7 to 71 months of age) for calendar years 2012–2013 combined, compared with age and season-matched reference cohorts before NVP introduction. The overall relative rate reduction in PCV10-type IPD was 56% (95%CI 24 to 76). The overall reduction in IPD was 48% (95%CI 18 to 69) and the absolute rate reduction 7.9 (95%CI 3 to 13) cases per 100,000 children per year. The number of CSF culture positive meningitis cases was too small for analysis.

**Table 2:** Rates of IPD and the corresponding rate reductions in the unvaccinated older target cohort vs. reference cohorts.

Serogroup	Incidence/100 000 person-years (N)		Relative rate reduction, % (95% CI)	Absolute rate reduction (95% CI)
	Reference cohorts^1^ ^)^	Target cohort^2^ ^)^	Target cohort vs. Reference cohorts combined	Target cohort vs. Reference cohorts combined
	2005–2006 & 2007–2008	2012–2013		
PCV10 serotypes^3^ ^)^	12.8 (27+44)	5.7 (14)	56 (24, 76)	7.1 (3, 11)
PCV10-related serotypes^4^ ^)^	1.8 (5+5)	2.4 (6)	-35 (-263, 54)	-0.6 (-3, 2)
6A	0.9 (1+4)	1.6 (4)	-80 (-579, 56)	-0.7 (-3, 1)
19A	0.7 (3+1)	0.8 (2)	-12 (-476, 84)	-0.1 (-1, 1)
Non-PCV10 serotypes^5^ ^)^	1.1 (3+3)	0.4 (1)	63 (-119, 98)	0.7 (-1, 2)
3	0.2 (1+0)	0.4 (1)	-125 (-17534, 97)	-0.2 (-1, 1)
22F	0.2 (0+1)	0.0 (0)	100 (-8661, 100)	0.2 (0, 1)
Undefined^6^ ^)^	0.7 (1+3)	0.0 (0)	100 (-240, 100)	0.7 (0, 1)
Any culture confirmed IPD	16.4 (36+55)	8.5 (21)	48 (18, 69)	7.9 (3, 13)

^1)^ Follow-up years in the two reference cohorts 273352+281012, age 19–71 months, born Jan’02-May’04 or Jan’04-May’06

^2)^ Follow-up years in the target cohort 246773, age 19–71 months, born Jan’08-May’10

^3)^ In these data: 4, 6B, 7F, 9V, 14, 18C, 19F, 23F

^4)^ In these data: 6A, 7, 9N, 19A

^5)^ In these data: 3, 8, 11A, 15B, 15C, 22F, 33, 38

^6)^ No isolate available

## Discussion

During the first three years after NVP introduction, we observed a significant reduction in rates of overall, PCV10-type and PCV10-related type IPD with estimates ranging from 70% to 100% among children eligible for PCV10 vaccination. As approximately 200,000 children had been vaccinated with PCV10 by the end of 2013, these data provide convincing evidence for the effectiveness against vaccine-serotype IPD. We also observed a reduction in IPD among unvaccinated older children already one year after PCV10 introduction.

Our findings are consistent with the first results from an observational study in Quebec, Canada [6,7], suggesting significant reductions in IPD rates in birth cohorts vaccinated with PCV10 compared with those vaccinated with PCV7. However, the ability to quantify the specific PCV10 vaccine effect in that study was limited because of a previous PCV7 vaccination program. In addition, studies of PCV7 effects have reported substantial reductions in IPD rates in unvaccinated groups as result of herd protection [2,3]. In our study, the 50% reduction in overall IPD rates among unvaccinated children 19 to 71 months of age, a year after PCV10 introduction, suggests that PCV10 also elicits herd protection against IPD. To our knowledge, this is the first nation-wide population-based study to document the direct and indirect effects of routine PCV10 vaccination among vaccine-eligible and unvaccinated children.

Previously, increases in non-vaccine type IPD, particularly due to serotype 19A, have decreased the overall public health impact seen after routine PCV7 use [2,3]. In vaccine-eligible children, we observed a significant reduction in PCV10-related IPD including 6A and 19A, suggesting that PCV10 may also provide cross-protection against vaccine related serotypes. The latter finding is consistent with a recent case-control study in Brazil reporting significant effectiveness of PCV10 against serotype 19A [8]. However, the reduced impact on PCV10-related type IPD in 2013, both in vaccine-eligible as well as in unvaccinated children, gave first indications of possible decrease in vaccine effectiveness. In addition, an increasing trend in the incidence of non-PCV10 types suggested the development of replacement, although the absolute rates remained low. These observations warrant continuing monitoring of the potential shifts in the serotype distribution after introduction of PCV10.

Population-based surveillance data on long-term trends in serotype distribution are the key in interpreting post-vaccine program changes. Some of the observed changes may be attributable to changes in clinical practice or in surveillance methodology or quality. The strengths in evaluating the validity of our findings include high data completeness (almost 100% of isolates available for serotyping) and a well-established and consistent reporting in our population-based nationwide surveillance system. In addition, record linkage using the Personal Identity Code allowed us to separate vaccine-eligible and unvaccinated birth cohorts for evaluation of the direct and indirect effects. However, the number of IPD cases in our study was relatively small reducing the ability to make firm conclusions, particularly about individual serotypes.

Before-after comparisons are prone to bias due to secular trends. In Finland, secular trends have been identified with specific serotypes during the pre-PCV10 era, including an increasing trend in serotype 14 [16] and increasing rates of overall IPD before PCV10 program implementation. The temporal increase and higher regional rates were associated with higher blood culture rates over time and by region [17]. The potential increasing trend in the baseline IPD rate suggests that our reported estimates for reduction may be conservative.

Studies evaluating the population effects of PCVs have demonstrated that full characterization of the total impact of PCV programs in the population, especially the indirect effects, requires several years of follow-up [7, 18]. Therefore, due to short time period since NVP introduction, investigation of the indirect impact of routine PCV10 vaccination is challenging. On the basis of studies of nasopharyngeal colonization [19–21], children are considered the primary reservoir for transmission of pneumococcus. Thus the most likely age-groups for early detection of indirect effects are groups in close contact with NVP-eligible children, such as children attending the same daycare. We therefore focused on unvaccinated children to assess potential emerging indirect impact. Our results showing significant effectiveness of PCV10 against IPD also in non-vaccinated children are consistent with the results of a randomized-controlled carriage study in unvaccinated older siblings of PCV10-vaccinated children [22].

In conclusion, our national study adds to the evidence that routine PCV10 vaccination program’s effects on culture-confirmed IPD are similar to those previously reported for PCV7 and PCV13 programs [2, 23–28]. Further follow-up is needed to fully characterize the extent of serotype replacement in IPD after NVP, both in vaccinated and unvaccinated populations. Furthermore, the estimation of the full public health impact of the PCV programs warrant evaluating the impact on other disease syndromes preventable by PCV vaccination, such as clinically suspected IPD without laboratory-confirmation [13], pneumonia and upper respiratory diseases [29].

## Supporting Information

S1 Table Rates of IPD and the corresponding rate reductions in the unvaccinated cohort vs reference cohorts in years 2004, 2006, and 2011. (DOC)

S2 Table Rates of IPD and the corresponding rate reductions in the unvaccinated cohort vs reference cohorts in years 2005, 2007, and 2012. (DOC)

S3 Table Rates of IPD and the corresponding rate reductions in the unvaccinated cohort vs reference cohorts in years 2006, 2008, and 2013. (DOC)
