# Supplementary material for: Impact of Ten-Valent Pneumococcal Conjugate Vaccination on Invasive Pneumococcal Disease in Finnish Children – A Population-Based Study
Source: PLoS One. 2015 Mar 17;10(3):e0120290. doi: 10.1371/journal.pone.0120290 (PMC4364013; doi:10.1371/journal.pone.0120290)
Supplement: S1 Table — (DOC) [file pone.0120290.s001.doc]

Supplement Table 1: Rates of IPD and the corresponding rate reductions in the unvaccinated cohort vs reference cohorts in years 2004, 2006, and 2011.

| Serotype group | Incidence/100 000 person-years (N) | | Relative rate reduction (95% CI) | Absolute rate reduction (95% CI) |
| --- | --- | --- | --- | --- |
|  | Reference cohorts | Target cohort |  |  |
|  | 2004&2006^1)^ | 2011^2)^ | 2011 vs. 2004&2006 | 2011 vs. 2004&2006 |
| PCV10 serotypes^3)^ | 34.1 (39+55) | 28.6 (35) | 16 (-23, 4) | 5.4 (-6, 17) |
| PCV10-related serotypes^4)^ | 8.3 (16+7) | 6.5 (8) | 22 (-68, 67) | 1.8 (-4, 8) |
| 6A | 1.8 (3+2) | 0.0 (0) | 100 (-146, 100) | 1.8 (0, 3) |
| 19A | 5.1 (10+4) | 6.5 (8) | -29 (-201, 49) | -1.5 (-7, 4) |
| Non-PCV10 serotypes^5)^ | 2.2 (3+3) | 4.9 (6) | -126 (-621, 29) | -2.7 (-7, 2) |
| 3 | 0.7 (1+1) | 0.8 (1) | -13 (-1078, 95) | -0.1 (-2, 2) |
| 22F | 0.4 (1+0) | 1.6 (2) | -351 (-Inf, 77) | -1.3 (-4, 1) |
| Undefined^6)^ | 2.2 (5+1) | 0.0 (0) | 100 (-92, 100) | 2.2 (0, 4) |
| Any culture confirmed IPD | 46.7 (63+66) | 40.1 (49) | 14 (-18, 39) | 6.7 (-7, 21) |

^1)^ Follow-up years 136,174+139,820, age 7-48 months, born Jan’01-May’03 or Jan’03-May’05

^2)^ Follow-up years 122,331, age 7-48 months, born Jan’08-May’10

^3)^ In these data: 4, 6B, 7F, 9V, 14, 18C, 19F, 23F

^4)^ In these data: 6, 6A, 7, 9N, 19A

^5)^ In these data: 3, 33, 15B, 15C, 22F, 38

^6)^ No isolate available or serotype pending
