# Supplementary material for: Impact of Ten-Valent Pneumococcal Conjugate Vaccination on Invasive Pneumococcal Disease in Finnish Children – A Population-Based Study
Source: PLoS One. 2015 Mar 17;10(3):e0120290. doi: 10.1371/journal.pone.0120290 (PMC4364013; doi:10.1371/journal.pone.0120290)
Supplement: S2 Table — (DOC) [file pone.0120290.s002.doc]

Supplement Table 2. Rates of IPD and the corresponding rate reductions in the unvaccinated cohort vs reference cohorts in years 2005, 2007, and 2012.

| Serotype group | Incidence/100 000 person-years (N) | | Relative rate reduction (95% CI) | Absolute rate reduction (95% CI) |
| --- | --- | --- | --- | --- |
|  | Reference cohorts | Target cohort |  |  |
|  | 2005&2007^1)^ | 2012^2)^ | 2012 vs. 2005&2007 | 2012 vs. 2005&2007 |
| PCV10 serotypes^3)^ | 19.5 (20+34) | 8.9 (11) | 54 (16, 77) | 10.6 (3, 18) |
| PCV10-related serotypes^4)^ | 1.8 (2+3) | 1.6 (2) | 10 (-317, 87) | 0.2 (-3, 3) |
| 6A | 1.1 (1+2) | 1.6 (2) | -50 (-805, 80) | -0.5 (-3, 2) |
| 19A | 0.4 (0+1) | 0.0 (0) | 100 (-8671, 100) | 0.4 (-0, 1) |
| Non-PCV10 serotypes^5)^ | 1.1 (2+1) | 0.0 (0) | 100 (-444, 100) | 1.1 (-0, 2) |
| 3 | 0.0 (0+0) | 0.0 (0) | - | 0.0 (-0, 0) |
| 22F | 0.0 (0+0) | 0.0 (0) | - | 0.0 (-0, 0) |
| Undefined^6)^ | 1.1 (1+2) | 0.0 (0) | 100 (-444, 100) | 1.1 (-0, 2) |
| Any culture confirmed IPD | 23.5 (25+40) | 10.6 (13) | 55 (21, 76) | 12.9 (5, 21) |

^1)^ Follow-up years 136,538+140, 226, age 19-60 months, born Jan’01-May’03 or Jan’03-May’05

^2)^ Follow-up years 123, 068, age 19-60 months, born Jan’08-May’10

^3)^ In these data: 4, 6B, 9V, 14, 18C, 19F, 23F

^4)^ In these data: 6A, 9N, 19A

^5)^ In these data: 8, 15B, 15C

^6)^ No isolate available or serotype pending
