# Supplementary material for: Impact of Ten-Valent Pneumococcal Conjugate Vaccination on Invasive Pneumococcal Disease in Finnish Children – A Population-Based Study
Source: PLoS One. 2015 Mar 17;10(3):e0120290. doi: 10.1371/journal.pone.0120290 (PMC4364013; doi:10.1371/journal.pone.0120290)
Supplement: S3 Table — (DOC) [file pone.0120290.s003.doc]

Supplement Table 3. Rates of IPD and the corresponding rate reductions in the unvaccinated cohort vs reference cohorts in years 2006, 2008, and 2013.

| Serotype group | Incidence/100 000 person-years (N) | | Relative rate reduction (95% CI) | Absolute rate reduction (95% CI) |
| --- | --- | --- | --- | --- |
|  | Reference cohorts | Target cohort |  |  |
|  | 2006&2008^1)^ | 2013^2)^ | 2013 vs. 2006&2008 | 2013 vs. 2006&2008 |
| PCV10 serotypes^3)^ | 6.1 (7+10) | 2.4 (3) | 60 (-18, 91) | 3.7 (-0, 8) |
| PCV10-related serotypes^4)^ | 1.8 (3+2) | 3.2 (4) | -80 (-579, 56) | -1.4 (-5, 2) |
| 6A | 0.7 (0+2) | 1.6 (2) | -124 (-2996, 84) | -0.9 (-3, 2) |
| 19A | 1.1 (3+0) | 1.6 (2) | -50 (-1206, 88) | -0.5 (-3, 2) |
| Non-PCV10 serotypes^5)^ | 1.1 (1+2) | 0.8 (1) | 25 (-484, 96) | 0.3 (-2, 2) |
| 3 | 0.4 (1+0) | 0.8 (1) | -124 (-17515, 97) | -0.4 (-2, 1) |
| 22F | 0.4 (0+1) | 0.0 (0) | 100 (-8652, 100) | 0.4 (-0, 1) |
| Undefined^6)^ | 0.4 (0+1) | 0.0 (0) | 100 (-8652, 100) | 0.4 (-0, 1) |
| Any culture confirmed IPD | 9.4 (11+15) | 6.5 (8) | 31 (-46, 71) | 2.9 (-3, 9) |

^1)^ Follow-up years 136,814+140,786, age 31-72 months, born Jan’01-May’03 or Jan’03-May’05

^2)^ Follow-up years 123,705, age 31-72 months, born Jan’08-May’10

^3)^ In these data: 6B, 7F, 9V, 14, 18C, 23F

^4)^ In these data: 6A, 19A

^5)^ In these data: 3, 11A, 22F

^6)^ No isolate available or serotype pending
